# Photophysical Properties of Spirobifluorene-Based *o*-Carboranyl Compounds Altered by Structurally Rotating the Carborane Cages

**DOI:** 10.3390/molecules24224135

**Published:** 2019-11-15

**Authors:** Seonah Kim, Hyunhee So, Ji Hye Lee, Hyonseok Hwang, Hyoshik Kwon, Myung Hwan Park, Kang Mun Lee

**Affiliations:** 1 Department of Chemistry, Institute for Molecular Science and Fusion Technology, Kangwon National University, Chuncheon 24341, Korea; 2 Department of Chemistry Education, Chungbuk National University, Cheongju 28644, Korea

**Keywords:** *o*-carborane, intramolecular charge transfer, radiative decay, rotation

## Abstract

9,9′-Spirobifluorene-based *o*-carboranyl compounds **C1** and **C2** were prepared and fully characterized by multinuclear nuclear magnetic resonance (NMR) spectroscopy and elemental analysis. The solid-state structure of **C1** was also determined by single-crystal X-ray diffractometry. The two carboranyl compounds display major absorption bands that are assigned to *π*−π* transitions involving their spirobifluorene groups, as well as weak intramolecular charge-transfer (ICT) transitions between the *o*-carboranes and their spirobifluorene groups. While **C1** only exhibited high-energy emissions (λ_em_ = ca. 350 nm) in THF at 298 K due to locally excited (LE) states assignable to *π*−π* transitions involving the spirobifluorene group alone, a remarkable emission in the low-energy region was observed in the rigid state, such as in THF at 77 K or the film state. Furthermore, **C2** displays intense dual emissive patterns in both high- and low-energy regions in all states. Electronic transitions that were calculated by time-dependent-DFT (TD-DFT) for each compound based on ground (S_0_) and first-excited (S_1_) state optimized structures clearly verify that the low-energy emissions are due to ICT-based radiative decays. Calculated energy barriers that are based on the relative energies associated with changes in the dihedral angle around the *o*-carborane cages in **C1** and **C2** clearly reveal that the *o*-carborane cage in **C1** rotates more freely than that in **C2**. All of the molecular features indicate that ICT-based radiative decay is only available to the rigid state in the absence of structural fluctuations, in particular the free-rotation of the *o*-carborane cage.

## 1. Introduction

To date, *closo*-*ortho*-carborane (1,2-C_2_B_10_H_12_) derivatives, which are well-known icosahedral boron-cluster compounds, have been widely studied for their interesting optoelectronic properties, which are based on a variety of organic/organometallic luminophores [1,2,3,4,5,6,7,8,9,10,11,12,13,14,15,16,17,18,19,20,21,22,23,24,25,26,27,28]. Indeed, these desirable electronic properties are due to the unique characteristics of the *o*-carborane unit, such as its strongly electron-deficient nature, as well as its highly polarizable σ-aromaticity. In addition, these properties facilitate the formation of various donor‒acceptor systems, leading to intriguing intramolecular charge-transfer (ICT) transitions between numerous *π*-conjugated aromatic groups and *o*-carborane cages [29,30,31,32,33,34,35,36,37,38,39,40,41,42,43,44,45,46,47,48,49,50,51,52,53,54,55,56,57,58,59,60,61,62]. Hence, these features have become ultimate sources for generating specific luminescence behavior in a variety of *o*-carborane-based compounds. Importantly, the ICT-transition-based radiative mechanism that was observed in an *o*-carborane-incorporated system can be fine-tuned through strategic approaches, such as structurally varying its molecular geometry and the introduction of appropriate electron donors. As representative examples, the *C*-diazaboryl-*o*-carboranyl compounds that were reported by Fox and co-workers show dual emissive features that are characterized as locally excited (LE) high-energy states and low-energy ICT states [32]. These properties are the results of changes in the molecular structure and the regulation of the free rotations of the aromatic groups bound to the *o*-carboranyl units. In addition, various *o*-carborane-containing donor-acceptor systems exhibit intriguing photophysical properties through twisted intramolecular charge-transfer (TICT) processes [43,44,45,46,47,48,49,50,51,52,53]. Along with these previous studies, our group reported significant changes in radiative ICT mechanisms by modulating the planarities of several phenylene groups that were substituted on *o*-carborane cages [60,61,62]. Accordingly, we clearly demonstrated that the appropriate combination of molecular geometry and donor plays a key role in implementing promising optoelectronic features.

**Figure 1:**
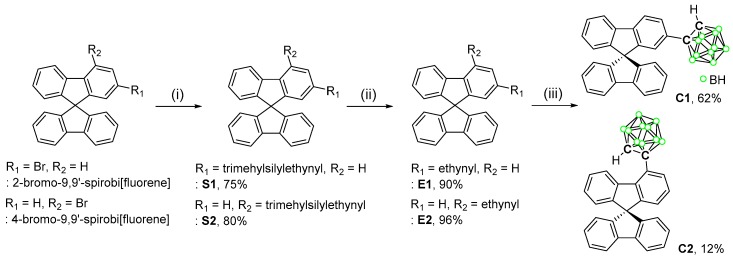
Synthesis routes to the spirobifluorene-based *o*-carboranyl complexes **C1** and **C2**. Reaction conditions: (i) Ethynyltrimethylsilane, CuI, Pd(PPh_3_)_2_Cl_2_, NEt_3_/toluene, r.t., 24 h. (ii) K_2_CO_3_, methanol r.t., 2 h. (iii) B_10_H_14_, Et_2_S, toluene, 110 °C, 72 h.

In this context, we selected 9,9′-spirobifluorene as the donor for attachment to the *o*-carborane in order to investigate the relationship between geometry and optoelectronic behavior in more detail. 9,9′-Spirobifluorene derivatives have been extensively used as prominent versatile materials [63] in a variety of optoelectronic applications, such as organic light-emitting diodes (OLEDs) [64,65,66,67,68,69] and photovoltaic cells [70,71,72]. Herein, we report two spirobifluorene-based *o*-carboranyl compounds as novel donor‒acceptor systems (Figure 1, **C1** and **C2**). The synthesis procedures, characterization data, and detailed photophysical properties are presented in conjunction with computational results.

## 2. Materials and Methods

### 2.1. General Considerations

All of the operations were performed in an inert nitrogen atmosphere while using standard Schlenk and glove box techniques. Anhydrous-grade solvents (toluene, triethylamine, and tetrahydrofuran (THF); Aldrich) were dried by passing through an activated alumina column and stored over activated molecular sieves (5 Å). Spectrophotometric-grade solvents (THF, toluene, dichloromethane (DCM), *n*-hexane, methanol, and ethanol) were used as received from Alfa Aesar. Commercial reagents and solvents for purification were used as received from Aldrich (bis(triphenylphosphine)palladium(II) dichloride (Pd(PPh_3_)_2_Cl_2_), copper(I) iodide (CuI), diethyl sulfide (Et_2_S), ethynyltrimethylsilane, *n*-tetrabutylammonium fluoride (TBAF), potassium carbonate (K_2_CO_3_), and poly(methyl methacrylate) (PMMA)), Alfa Aesar (decaborane (B_10_H_14_)), or TCI (2-bromo-9,9′-spirobi [9H-fluorene] and 4-bromo-9,9′-spirobi [9H-fluorene]). CDCl_3_ (purchased from Cambridge Isotope Laboratories) was used after drying over activated molecular sieves (5 Å). Nuclear magnetic resonance (NMR) spectra of all the compounds were recorded on a Bruker Avance 400 spectrometer (400.13 MHz for ^1^H, 100.62 MHz for ^13^C, and 128.38 MHz for ^11^B) at ambient temperature. Chemical shifts are given in ppm and they are referenced against external Me_4_Si (^1^H and ^13^C) and BF_3_·Et_2_O (^11^B). Elemental analyses were performed on an EA3000 (Eurovector) analyzer in the Central Laboratory of Kangwon National University. UV/vis absorption and PL spectra were recorded on a Jasco V-530 and a HORIBA FluoroMax-4P spectrophotometer, respectively. Fluorescence-decay lifetimes (τ_obs_) were measured at the Central Laboratory of Kangwon National University while using a time-correlated single-photon counting (TCSPC) spectrometer (FLS920-EDINBURGH Instruments) that was equipped with a EPL-375 picosecond pulsed semiconductor diode laser as the excitation source, and a microchannel plate photomultiplier tube (MCP-PMT, 200‒850 nm) as the detector at 298 K. Absolute photoluminescence quantum yields (PLQYs, Φ_em_) were determined while using an absolute PL quantum yield spectrophotometer (FM-SPHERE, 3.2-inch internal integrating sphere on FluoroMax-4P, HORIBA) at 298 K.

### 2.2. Synthesis of (9,9-Spirobi[Fluoren]-2-Ylethynyl)Trimethylsilane (S1)

Triethylamine (20 mL) was added via cannula to a mixture of 2-bromo-9,9′-spirobi[9H-fluorene] (1.0 g, 2.5 mmol), copper iodide (20 mg), and Pd(PPh_3_)_2_Cl_2_ (78 mg) at room temperature. After stirring for 15 min., ethynyltrimethylsilane (1.8 mL, 5.0 mmol) was added and the reaction mixture was heated at 90 °C with stirring for 24 h. After cooling to room temperature, the volatiles were removed by rotary evaporation to afford a dark brown residue. The crude product was purified by column chromatography on silica (eluent: DCM/*n*-hexane = 1/10, *v*/*v*) to yield **S1** as a white solid, 0.78 g (yield = 74.9%). ^1^H NMR (CDCl_3_): δ 7.85 (d, *J* = 7.6 Hz, 2H), 7.82 (d, *J* = 7.6 Hz, 1H), 7.77 (m, 1H), 7.49 (dd, *J* = 7.9, 1.5 Hz, 1H), 7.37 (td, *J* = 7.6, 1.0 Hz, 3H), 7.12 (td, *J* = 7.5, 1.0 Hz, 3H), 6.84 (m, 1H), 6.72 (dd, *J* = 7.6, 4.6 Hz, 3H), and 0.16 (s, 9H, Si(C*H*_3_)_3_). ^13^C NMR (CDCl_3_): δ 149.32, 148.82, 148.31, 142.28, 141.93, 141.14, 132.04, 128.45, 128.02, 127.74, 124.24, 122.28, 120.44, 120.20, 119.93, 105.54 (acetylene-*C*), 94.46 (acetylene-*C*), 65.90 (spiro-*C*), and 0.06 (Si(*C*H_3_)_3_). Anal. Calcd. for C_30_H_24_Si: C, 87.33; H, 5.86. Found: C, 87.16; H, 5.85.

### 2.3. Synthesis of (9,9′-Spirobi[Fluoren]-4-Ylethynyl)Trimethylsilane (S2)

**S2** was prepared as a white solid (1.64 g; yield = 79.5%) in a procedure that was analogous to that used for **S1**, with 4-bromo-9,9′-spirobi[9H-fluorene] (2.0 g, 5.0 mmol), copper iodide (38 mg), Pd(PPh_3_)_2_Cl_2_ (154 mg), and ethynyltrimethylsilane (3.5 mL, 9.8 mmol). ^1^H NMR (CDCl_3_): δ 8.66 (d, *J* = 7.8 Hz, 1H), 7.84 (d, *J* = 7.6 Hz, 2H), 7.47 (d, *J* = 0.9 Hz, 1H), 7.38 (m, 3H), 7.13 (m, 3H), 7.03 (t, *J* = 7.6 Hz, 1H), 6.71 (m, 4H), 0.41 (s, 9H, Si(C*H*_3_)_3_). ^13^C NMR (CDCl_3_): δ 149.40, 149.12, 148.58, 141.99, 141.96, 141.47, 132.58, 128.41, 128.03, 127.94, 127.64, 127.30, 124.46, 124.15, 123.85, 123.13, 120.16, 116.59, 104.19 (acetylene-*C*), 99.52 (acetylene-*C*), 65.54 (spiro-*C*), 0.13. Anal. Calcd. for C_30_H_24_Si: C, 87.33; H, 5.86. Found: C, 87.20; H, 5.77.

### 2.4. Synthesis of 2-Ethynyl-9,9′-Spirobi[Fluorene] (E1)

K_2_CO_3_ (0.23 g, 2.0 mmol) was dissolved in methanol (80 mL) and added to a solution of **S1** (0.77 g, 1.86 mmol) in DCM (10 mL). After stirring for 2 h at room temperature, distilled water (100 mL) was slowly poured to the reaction mixture and further stirred for 10 min. The resulting mixture was treated with DCM (50 mL) and the organic layer was separated. The aqueous layer was further extracted with DCM (20 × 2 mL). The combined organic extracts were dried over MgSO_4_, filtered, and evaporated to dryness to afford a white residue. The crude product was purified by washing with methanol (10 mL) to yield **E1** as a white solid, 0.57 g (yield = 90.0%). ^1^H NMR (CDCl_3_): δ 7.84 (dd, *J* = 5.8, 2.3 Hz, 3H), 7.79 (d, *J* = 7.9 Hz, 1H), 7.51 (dt, *J* = 7.9, 1.2 Hz, 1H), 7.38 (t, *J* = 7.5 Hz, 3H), 7.13 (m, 3H), 6.86 (s, 1H), 6.73 (m, 3H), 2.96 (s, 1H, acetylene-*H*). ^13^C NMR (CDCl_3_): δ 149.28, 149.02, 148.21, 142.56, 141.92, 141.06, 132.09, 128.57, 128.04, 127.98, 124.30, 124.19, 121.21, 120.51, 120.23, 120.04, 84.06 (acetylene-*C*), 77.36 (acetylene-*C*), 65.88 (spiro-*C*). Anal. Calcd. for C_27_H_16_: C, 95.26; and, H, 4.74. Found: C, 94.89; H, 4.54.

### 2.5. Synthesis of 4-Ethynyl-9,9′-Spirobi[Fluorene] (E2)

**E2** was prepared as a white solid (0.65 g; yield = 95.5%) in a procedure analogous to that used for **E1**, with **S2** (0.83 g, 2.0 mmol) and K_2_CO_3_ (0.55 g, 4.0 mmol). ^1^H NMR (CDCl_3_): δ 8.64 (d, *J* = 7.8 Hz, 1H), 7.84 (d, *J* = 7.6 Hz, 2H), 7.49 (dd, *J* = 7.7, 0.9 Hz, 1H), 7.38 (m, 3H), 7.13 (dd, *J* = 15.0, 6.5 Hz, 3H), 7.05 (t, *J* = 7.6 Hz, 1H), 6.72 (m, 4H), 3.58 (s, 1H, acetylene-*H*). ^13^C NMR (CDCl_3_): δ 149.51, 149.06, 148.50, 142.21, 141.96, 141.23, 133.06, 128.55, 128.05, 127.99, 127.84, 127.36, 124.79, 124.16, 123.90, 123.07, 120.19, 115.51, 82.71(acetylene-*C*), 82.09 (acetylene-*C*), 65.53 (spiro-*C*). Anal. Calcd. for C_27_H_16_: C, 95.26; H, 4.74. Found: C, 95.11; H, 4.52.

### 2.6. Synthesis of C1

Excess Et_2_S (2.5 equiv.) was added at room temperature to a toluene solution (15 mL) of decaborane (B_10_H_14_, 1.53 mmol) and **E1** (0.40 g, 1.18 mmol). After heating to reflux, the reaction mixture was further stirred for 72 h. The solvent and volatiles were removed under vacuum and methanol (10 mL) was added. The resulting solid was filtered and re-dissolved in toluene. The solution was purified by passing through a basic alumina column and the solvent was removed *in vacuo*. The product was purified by column chromatography on basic alumina (eluent: ether/*n*-hexane = 1/5, *v*/*v*) to yield **C1** as a white solid, 0.34 g (yield = 62.2%). ^1^H NMR (CDCl_3_): δ 7.87 (d, *J* = 7.7 Hz, 2H), 7.83 (d, *J* = 7.7 Hz, 1H), 7.77 (d, *J* = 8.2 Hz, 1H), 7.56 (dd, *J* = 8.2, 2.0 Hz, 1H), 7.39 (dt, *J* = 13.1, 7.5 Hz, 3H), 7.13 (m, 3H), 6.73 (d, *J* = 1.8 Hz, 1H), 6.69 (t, *J* = 6.8 Hz, 3H), 3.77 (s, 1H, CB-*H*), 2.82–1.62 (br, 10H, CB-B*H*). ^13^C NMR (CDCl_3_): δ 149.71, 149.68, 147.83, 143.87, 141.89, 132.97, 132.96, 129.02, 128.25, 128.18, 128.12, 124.23, 124.07, 122.70, 120.69, 120.39, 120.18, 77.36 (CB-*C*), 66.07 (spiro-*C*), 60.27 (CB-*C*). ^11^B{^1^H} NMR (CDCl_3_): δ −2.21, −4.54, −9.10, −10.88, −11.30, −12.92. Anal. Calcd. for C_27_H_26_B_10_: C, 70.71; H, 5.71. Found: C, 70.66; H, 5.64.

### 2.7. Synthesis of C2

**C2** was prepared in a procedure analogous to that used for **C1** with decaborane (B_10_H_14_, 3.08 mmol), **E2** (1.08 g, 2.37 mmol), and Et_2_S (2.5 equiv.). The product was purified by column chromatography on basic alumina (eluent: DCM/*n*-hexane = 1/10, *v*/*v*) to yield **C2** as a white solid, 0.20 g (yield = 12.3%). ^1^H NMR (CDCl_3_): δ 8.30 (d, *J* = 8.4 Hz, 1H), 7.83 (d, *J* = 7.6 Hz, 2H), 7.70 (m, 1H), 7.37 (m, 3H), 7.03 (m, 3H), 7.05 (m, 1H), 6.74 (dd, *J* = 7.6, 1.1 Hz, 1H), 6.69 (dd, *J* = 7.4, 1.0 Hz, 1H), 6.64 (d, *J* = 7.6 Hz, 2H), 5.02 (s, 1H, CB-*H*), 2.56–1.36 (br, 10H, CB-B*H*). ^13^C NMR (CDCl_3_): δ 152.32, 149.84, 148.41, 141.91, 139.51, 137.76, 131.53, 130.32, 128.80, 128.25, 128.23, 127.87, 127.79, 125.83, 125.26, 125.08, 123.97, 120.36, 77.76 (CB-*C*), 65.78 (spiro-*C*) 59.85 (CB-*C*). ^11^B{^1^H} NMR (CDCl_3_): δ −2.19, −3.23, −7.94, −9.08, −12.55, −13.76. Anal. Calcd. for C_27_H_26_B_10_: C, 70.71; H, 5.71. Found: C, 70.59; H, 5.61.

### 2.8. UV/Vis Absorption and Photoluminescence (PL) Experiments

Solution UV/Vis absorption and PL experiments were performed in degassed solvents (toluene, THF, and DCM) in 1-cm quartz cuvettes (30 μM) at 298 K. PL was also investigated in THF solution at 77 K and the film state (5 wt% doped on PMMA) for each *closo*-*o*-carborane compound on 1-mm-thick 1.5 × 1.5 cm quartz plates at 298 K. Absolute photoluminescence quantum yields (PLQYs, Φ_em_) for the solutions and films were obtained while using an absolute PL quantum yield spectrophotometer (FM-SPHERE, 3.2-inch internal integrating sphere on FluoroMax-4P, HORIBA) at 298 K.

### 2.9. X-Ray Crystallography

A single crystal of **C1** suitable for X-ray diffractometry was grown from a DCM/*n*-hexane mixture. The single crystal was coated with Paratone oil and then mounted in a glass capillary. Crystallographic experiments were performed on a Bruker D8 QUEST CCD area detector diffractometer with graphite-monochromated Mo-Kα radiation (*λ* = 0.71073 Å). Structures were solved by direct methods and all of the non-hydrogen atoms were subjected to anisotropic refinement while using the full-matrix least-squares method on *F*^2^ in the SHELXTL/PC package to obtain X-ray crystallographic data in CIF format (CCDC 1947010). Hydrogen atoms on the carbon and boron atoms were placed at their geometrically calculated positions and refined as riding on the corresponding carbon and boron atoms with isotropic thermal parameters. Detailed crystallographic data are provided in Tables S1 and S2

### 2.10. Computational Studies

The geometries of **C1** and **C2** in their ground (S_0_) and first-excited (S_1_) states in THF were optimized at the B3LYP/6-31G(d,p) [73] level of theory. Vertical excitation energies at the optimized ground- and first-excited-state geometries were calculated while using the time-dependent density functional theory (TD-DFT) method [74] at the same level of theory. Solvent effects were included while using the conductor-like polarizable continuum model (C-PCM) [75,76]. We constructed one dimensional potential energy surfaces (PESs) as function of dihedral angle (Ψ = C1‒C2‒C3‒C4) by rotating the *o*-carborane cage, *vide infra* (Figure 5) in the 0–180° range in 30° steps in order to determine the most stable geometries. Among the 49 initial conformations for each compound, conformations that show unphysical atomic overlaps were excluded from further geometry optimization. The dihedral angle was fixed, while the other geometrical variables were fully relaxed during geometry optimizations and energy calculations of the resultant initial conformations. All geometry optimizations and energy calculations were performed while using the Gaussian 16 program [77]. The frequency-checks for all of the optimized structures were performed and no imaginary frequency was confirmed. The percent contribution of a group in a molecule to each molecular orbital was calculated while using the GaussSum 3.0 program [78]. GaussView 6 was used to visualize molecular properties [79].

## 3. Results and Discussion

### 3.1. Synthesis and Characterization

**Figure 2:**
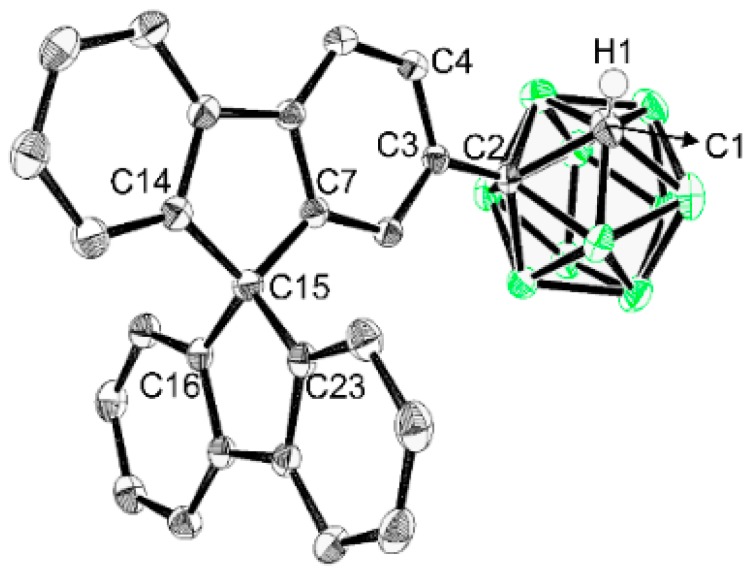
X-ray crystal structure of **C1** (50% thermal ellipsoids). H atoms are omitted for clarity.

Figure 1 shows the overall routes for the syntheses of the spirobifluorene-based carboranyl compounds **C1** and **C2**, with *o*-carborane cages substituted with fluorene moieties at their C2 and C4 positions, respectively. The Sonogashira-coupling reaction between ethynyltrimethylsilane and 2- or 4-bromo-9,9′-spirobifluorene produced (9,9′-spirobi[fluoren]-2-ylethynyl)trimethylsilane (**S1**) and (9,9′-spirobi[fluoren]-4-ylethynyl)trimethylsilane (**S2**) in high yields (75% for **S1** and 80% for **S2**). The protonated compounds **E1** and **E2** were obtained by the treatment of **S1** and **S2** with a weak base (K_2_CO_3_), after which the 2- and 4-*o*-carborane-substituted spirobifluorenes **C1** and **C2** were respectively prepared from **E1** and **E2** while using decaborane-promoted (B_10_H_14_-promoted) cage-forming reactions in the presence of Et_2_S (Figure 1) [61,62,63]. All of the compounds were fully characterized by multinuclear (^1^H, ^13^C, and ^11^B{^1^H}) NMR spectroscopy (Figures S1–S6) and elemental analysis. The ^1^H and ^13^C NMR spectra of **C1** and **C2** show resonances that correspond to the spirobifluorene moieties as well as the *o*-carboranyl groups, as expected. In particular, the peaks at ~66 ppm that were observed in the ^13^C NMR spectra correspond to the carbon atoms of the tetragonal centers of the spirobifluorene moieties. In addition, two signals are observed at around 77 and 60 ppm, which are assignable to the C atoms of the *o*-carboranyl groups. Six broad ^11^B NMR signals that were observed between −2 and −13 ppm clearly confirm the presence of the *closo*-carborane cages in **C1** and **C2**. Furthermore, the solid-state molecular structure of **C1** was determined by single-crystal X-ray diffractometry, as shown in Figure 2; detailed data are available in Table S1, while selected bond lengths and angles are listed in Table S2. The structure clearly exhibits a tetrahedral carbon center (average bond angle at C15 = 109.57°, Table S2) in the 9,9′-spirobifluorene moiety that was attached at the C2 position of the *o*-carborane cage.

### 3.2. Photophysical Properties

**Figure 3:**
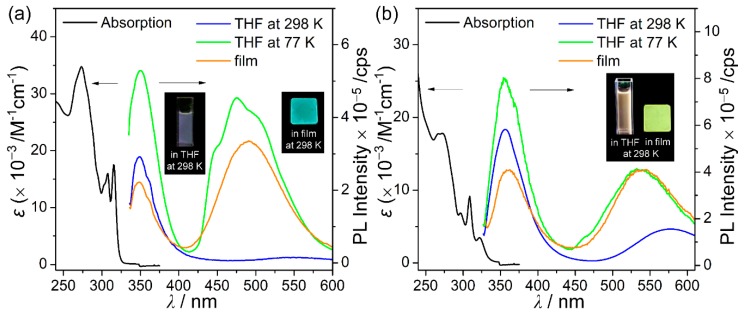
UV-vis absorption and photoluminescence (PL) spectra (λ_ex_ = 310 nm for **C1** and 322 nm for **C2**) of (**a**) **C1** and (**b**) **C2**. Black: absorption spectra in tetrahydrofuran (THF) (30 μM), blue: PL spectra in THF (30 μM) at 298 K, green: PL spectra in THF (30 μM) at 77 K, and orange: PL spectra in films (5 wt% doped with poly(methyl methacrylate) (PMMA)) at 298 K. The insets show the emission color of each state when irradiated with a hand-held UV lamp (λ_ex_ = 365 nm).

The photophysical properties of the spirobifluorene-based *o*-carboranyl compounds **C1** and **C2** were investigated by UV/Vis-absorption and PL spectroscopies (Figure 3 and Table 1). **C1** and **C2** display major absorption bands in the 270–322 nm region with structureless vibronic features. These bands are assigned to spin-allowed *π*−π* local transitions on each spirobifluorene moiety and typical intramolecular charge-transfer (ICT) transitions between each *o*-carborane unit and spirobifluorene group (see TD-DFT results, *vide infra*). The emissive properties of both compounds were examined by PL experiments under a variety of conditions (Figure 3 and Table 1). The emission spectrum of **C1** in THF at 298 K exhibits an intense emission in the high-energy region at λ_em_ = 349 nm due to *π*−π* transitions that are based on the spirobifluorene moiety. The emission band was consistently maintained in a variety of solvents of different polarity (λ_em_ = 349‒350 nm, Table 1 and Figure S7a), which further confirms the locally excited (LE) emission. Meanwhile, the PL spectra of **C1** in the rigid state (THF at 77 K and the film state (5 wt% doped on poly(methyl methacrylate) (PMMA)) show intriguing dual emissive patterns, each with an intense high-energy LE emission and a significantly broad low-energy emission (λ_em_ = 476 nm in THF at 77 K and λ_em_ = 490 nm in the film) that tailed to 600 nm in the low-energy region. We conclude that these low-energy emissions are closely associated with intramolecular charge-transfer (ICT) transitions between the *o*-carborane cage and the spirobifluorene moiety based on the computational results (*vide infra*).

Interestingly, such dual emissive behavior is also observed in the PL spectra of **C2** under all conditions (in THF at 298 K and 77 K, and in the film state, Figure 3). The ICT character of the low-energy emission from **C2** was confirmed by the solvatochromism that was observed in various solvents of different polarity (Table 1 and Figure S8b). These interesting features suggest that the ICT-based radiative decay processes in **C1** and **C2** are significantly enhanced in rigid molecular states that effectively prevent structural fluctuations, such as the C−C bond variations that occur in *o*-carborane cages [8,35,55,61,62]. Indeed, the DFT-optimized structures of the ground (S_0_) and first-excited singlet (S_1_) states of both **C1** and **C2** confirm such structural fluctuations. The bond lengths (2.38 Å in **C1** and 2.45 Å in **C2**) in their S_1_-optimized structures are much longer than those (≈1.7 Å) of their S_0_-optimized structures. In particular, the bond length in **C1** in the S_1_ state is also considerably longer than that (1.662 Å, Table S2) determined by X-ray diffractometry. Importantly, the ICT-based emission in THF at 298 K was only observed for **C2**, which indicated that **C2** undergoes smaller structural variations in solution than **C1**, which is supported by additional relative free-energy calculations in which the dihedral angles around the *o*-carboranyl cages in **C1** and **C2** were altered (*vide infra*).

The absolute PL quantum efficiencies (Φ_em_) and decay lifetimes (τ_obs_) of **C1** and **C2** were determined in dilute THF solutions and film states at 298 K to gain insight into the relationship between structure and the radiative decay mechanism for the ICT-based emission (Table 1 and Figures S8 and S9). The emission decay lifetimes, which were measured to be 1.2‒1.5 ns for both compounds, reveal that these emissions are fluorescent. The Φ_em_ values of **C2** in THF at 298 K and the film were determined to be 7% and 41%, respectively; however, the value for **C1** could only be measured in the film state, where it was found to be 2%. In the film state, the radiative decay constant (*k*_r_, 2.7 × 10^8^ s^−1^, Table 1) for the ICT-based emission from **C2**, as calculated from the Φ_em_ and τ_obs_ values, is five-times larger than that (0.5 × 10^8^ s^−1^) in THF, whereas the nonradiative decay constant (*k*_nr_) was calculated to be 3.9 × 10^8^ s^−1^, which is much smaller than that (6.6 × 10^8^ s^−1^) determined in THF. These results reveal that restricting structural variations can lead to more radiative decay and fewer non-radiative decay ICT transitions. Interestingly, the *k*_r_ value (1.7 × 10^7^ s^−1^) of **C1** in the film is significantly (15-times) lower than that of **C2**, but its *k*_nr_ value (8.3 × 10^8^ s^−1^) is much larger than that of **C2**. These findings demonstrate that structural fluctuations in **C1**, especially the free rotation of the *o*-carboranyl cage, are more severe than those in **C2**.

### 3.3. Computational Chemistry and Orbital Analyses

**Figure 4:**
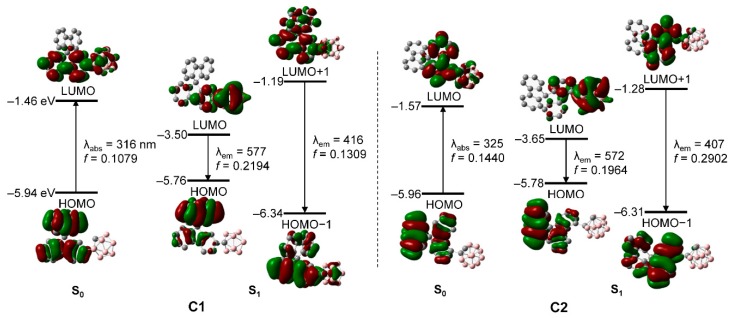
Frontier molecular orbitals of **C1** and **C2** in their ground states (S_0_) and first-excited singlet states (S_1_) and their relative energies calculated by DFT (isovalue = 0.04 a.u.). The transition energy (in nm) was calculated at the TD-B3LYP/6-31G(d) level of theory.

To elucidate the nature of the electronic transitions and to analyze the orbitals in **C1** and **C2**, their *S*_0_- and *S*_1_-optimized structures were subjected to TD-DFT calculations while using the B3LYP functional (Figure 4 and Table 2); the geometries were optimized from initial structures based on the X-ray crystal structure of **C1**. To include the effects of the THF solvent [75,76], a conductor-like polarizable continuum model was also used. The computational data for the *S*_0_ state show that HOMO → LUMO transitions are the lowest-energy electronic transitions in **C1** and **C2**. The HOMO of each compound is entirely localized on the bifluorene moiety (>99%, Tables S4 and S6), whereas the orbital contribution of the *o*-carborane unit to each LUMO is slightly higher, at >15%. These results indicate that the lowest-energy absorptions of **C1** and **C2** are attributable to the *π*−π* transitions in the bifluorene moieties, with minor contributions from ICT transitions between the *o*-carborane and fluorene groups. All of the calculated results based on the optimized *S*_0_ structures are in good agreement with the experimentally observed UV/Vis absorption spectra.

On the other hand, the calculated results for the *S*_1_ states of **C1** and **C2** show that the major transitions associated with the low-energy emissions involve both HOMO → LUMO and HOMO−1 → LUMO+1 transitions (Figure 4 and Table 2). While the LUMO of each compound is significantly localized in the *o*-carborane moiety (∼82%, Tables S4 and S6), each HOMO is predominantly located in the fluorene group (>99%). These results strongly suggest that the experimentally observed emissions in the low-energy regions mainly originate from ICT transitions between the *o*-carborane and aryl moieties. In addition, each HOMO−1 and LUMO+1 are mostly located on the bifluorene group (>92%, Tables S4 and S6), which strongly suggests that the intense emissions observed in the high-energy region centered at ~349 nm for **C1** and ~356 nm for **C2** originate from *π*−π* transitions in the bifluorene moieties; i.e., LE-based emissions. Consequently, the electronic transitions that occur in each *o*-carboranyl compound were precisely predicted while using computational methods.

### 3.4. DFT Energy-Barrier Calculations for Rotational Motion of the o-Carboranyl Cage

**Figure 5:**
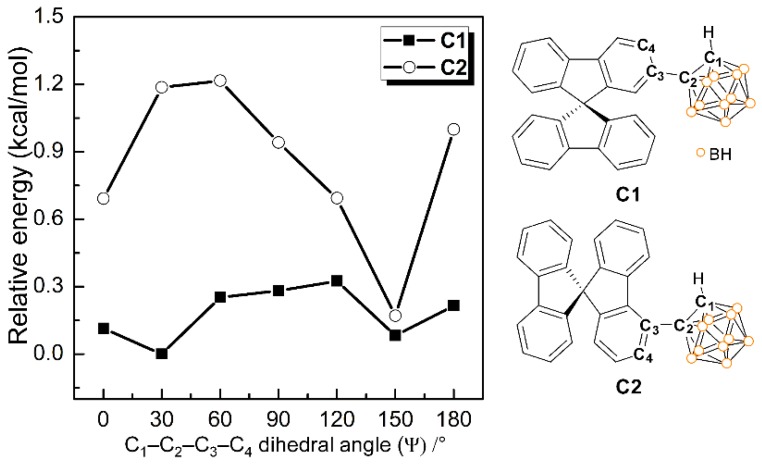
Relative energy diagrams (ΔE/kcal·mol^−1^) for **C1** and **C2**: relative energy as a function of dihedral angle in each ground (S_0_) state.

**Figure 6:**
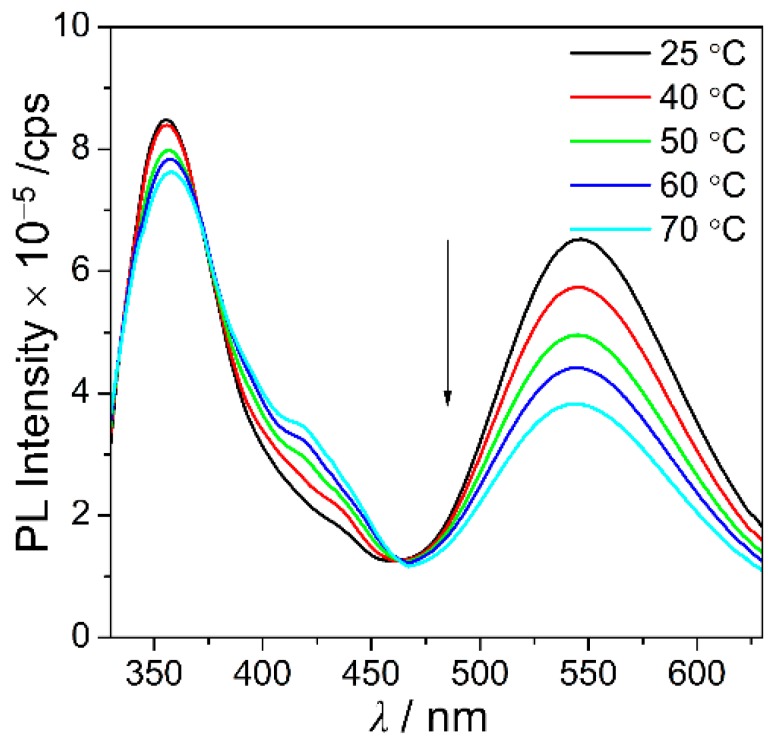
PL spectra of **C2** in toluene (30 μM, λ_ex_ = 322 nm) as functions of temperature.

The energies of **C1** and **C2** in their ground states (S_0_) were calculated as functions of the dihedral angles associated with their *o*-carboranyl cages (Ψ: C_1_‒C_2_‒C_3_‒C_4_, Figure 5) using the B3LYP functional and the 6-31G(d) basis set to gain insight into the relationship between the radiative mechanism for the ICT-based emissions observed for the spirobifluorenyl compound and the structure of its *o*-carboranyl moiety. The thermodynamic stability of each system is reported as the relative energy (ΔE/kcal·mol^−1^) against that of the S_0_-optimized structure of **C1** in THF (i.e., the thermal energy of the optimized structure of **C1** was assigned to be: E = 0 kcal·mol^−1^). The relative energy was calculated at each Ψ value in the 0–180° range in 30° steps, with the resulting energy diagrams for **C1** and **C2** displayed in Figure 5. The Ψ values for the S_0_-optimized structures of **C1** and **C2** were 30.2° and 152.4°, respectively, which correspond to their lowest relative energies. Importantly, the relative energy barrier for **C1** does not exceed 0.35 kcal·mol^−1^ over the entire Ψ range (Figure 5, squares), while the low energy region of **C2** is mainly centered at Ψ = 150° (Figure 6, circles). In addition, the energy gap between the minimum and maximum points for **C2** is nearly 1.2 kcal·mol^−1^. Based on the thermal energy (0.59 kcal·mol^−1^ calculated as *k*_B_T (*k*_B_ = Boltzmann constant, 1.98 cal·K^−1^·mol^−1^)) at 298 K, these results strongly indicate that the *o*-carborane cage in **C1** can freely rotate at room temperature, whereas the *o*-carborane cage in **C2** is fixed in all states. Consequently, these results verify that the structural rigidity of **C2** induces efficient radiative decay that is based on ICT transition associated with the *o*-carborane moiety. Furthermore, the ICT-based emission from **C2** in the solution state (30 μM in toluene) was observed to gradually decrease with increasing temperature, while the LE emission was essentially unchanged. These findings also confirm that the structural changes associated with the *o*-carborane cages can distinctly turn off radiative decay that is based on ICT transitions (Figure 6).

## 4. Conclusions

We prepared and characterized two spirobifluorenyl *o*-carborane compounds, **C1** and **C2**, with the solid-state structure of **C1** determined by single-crystal X-ray diffractometry. In particular, **C2** showed clear and intense ICT-based emissions that involve the *o*-carborane moiety in all states; however, **C1** only displayed an LE-based emission that was attributable to *π*−π* transitions centered on the spirobifluorenyl group in solution at 298 K, while **C2** exhibited additional ICT-based emissions in the rigid state. Energy-barriers that were determined from calculated relative free-energies of rotated *o*-carborane cages in both **C1** and **C2** decisively show that the *o*-carborane cage in **C1** more freely rotates than that in **C2**. Consequently, all of the characterization and computational results definitively show that large structural variations involving the *o*-carborane cage, namely its free-rotation, hinder ICT-based radiative decay that is associated with the *o*-carborane. This finding provides new decisive evidence that clarifies the relationship between structure and the efficiency of the ICT-based radiative decay that is associated with the *o*-carborane.

## Figures and Tables

**Table 1:** Photophysical data for spirobifluorene-based *o*-carboranyl compounds **C1** and **C2**.

Compound	*λ*_abs_^1^/nm (ε × 10^−3^ M^−1^ cm^−1^)		*λ*_ex_/nm	*λ*_em_/nm				
				Tol ^2^	THF ^2^	DCM ^2^	77 K ^1^	film ^3^
**C1**	307 (15.7), 315 (17.5)		310	350	349	349	350, 476	349, 490
**C2**	309 (9.2), 322 (3.5)		322	356, 545	356, 577	356, 588	355, 545	359, 539
**Compound**	***Φ*_em_^4,5^**		**τ/ns ^5^**		***k*_r_^6^/× 10^8^ s^−1^**		***k*_nr_^7^/× 10^8^ s^−1^**	
	**THF ^2^**	**film ^2^**	**THF ^2^**	**film ^2^**	**THF ^2^**	**film ^2^**	**THF ^2^**	**film ^2^**
**C1**	- ^8^	0.02	- ^8^	1.2	-	0.17	-	8.3
**C2**	0.07	0.41	1.4	1.5	0.50	2.7	6.6	3.9

^1^*c* = 30 μM in THF. ^2^ *c* = 30 μM, observed at 298 K. ^3^ Measured in the film state (5 wt% doped on PMMA) at 298 K. ^4^ Absolute PL quantum yield. ^5^ Measured for the ICT-based emissive band. ^6^ *k*_r_ = *Φ*_em_/*τ*. ^7^ *k*_nr_ = *k*_r_(1/*Φ*_em_−1). ^8^ Not observed due to weak emission.

**Table 2:** Major low-energy electronic transitions in **C1** and **C2** involving their ground states (S_0_) and first-excited singlet states (S_1_) calculated at the TD-B3LYP/6-31G(d) level of theory.^1^

	State	*λ*_calc_/nm	*f*_calc_	Assignment
**C1**	S_0_	316.26	0.1079	HOMO → LUMO (97.7%)
	S_1_	576.80 415.74	0.2194 0.1309	HOMO → LUMO (99.8%) HOMO−1 → LUMO+1 (91.2%)
**C2**	S_0_	324.89	0.1440	HOMO → LUMO (91.7%)
	S_1_	571.55 407.40	0.1964 0.2902	HOMO → LUMO (98.6%) HOMO−1 → LUMO+1 (93.1%)

^1^ Singlet energies for vertical transitions were calculated while using optimized *S*_1_ geometries.

## Supplementary Materials

The supplementary materials are available online.
