# Supplementary material for: Photophysical Properties of Spirobifluorene-Based o-Carboranyl Compounds Altered by Structurally Rotating the Carborane Cages
Source: Molecules. 2019 Nov 15;24(22):4135. doi: 10.3390/molecules24224135 (PMC6891568; doi:10.3390/molecules24224135)
Supplement: Supplementary file 1 [file molecules-24-04135-s001.pdf]

Supplementary Materials

Photophysical Properties for Spirobifluorene based *o*-Carboranyl Compounds Altered by Structural Rotation of Carborane Cages

Seonah Kim[1,†], Hyunhee So[1,†], Ji Hye Lee[1], Hyonseok Hwang[1], Hyoshik Kwon,[2,*] Myung Hwan Park[2,*] and Kang Mun Lee[1,*]

[1] Department of Chemistry, Institute for Molecular Science and Fusion Technology, Kangwon National University, Chuncheon 24341, Republic of Korea, kangmunlee@kangwon.ac.kr (K.M.L.)

[2] Department of Chemistry Education, Chungbuk National University, Cheongju 28644, Republic of Korea, hskwon@chungbuk.ac.kr (H.K.); mhpark98@chungbuk.ac.kr (M.H.P.)

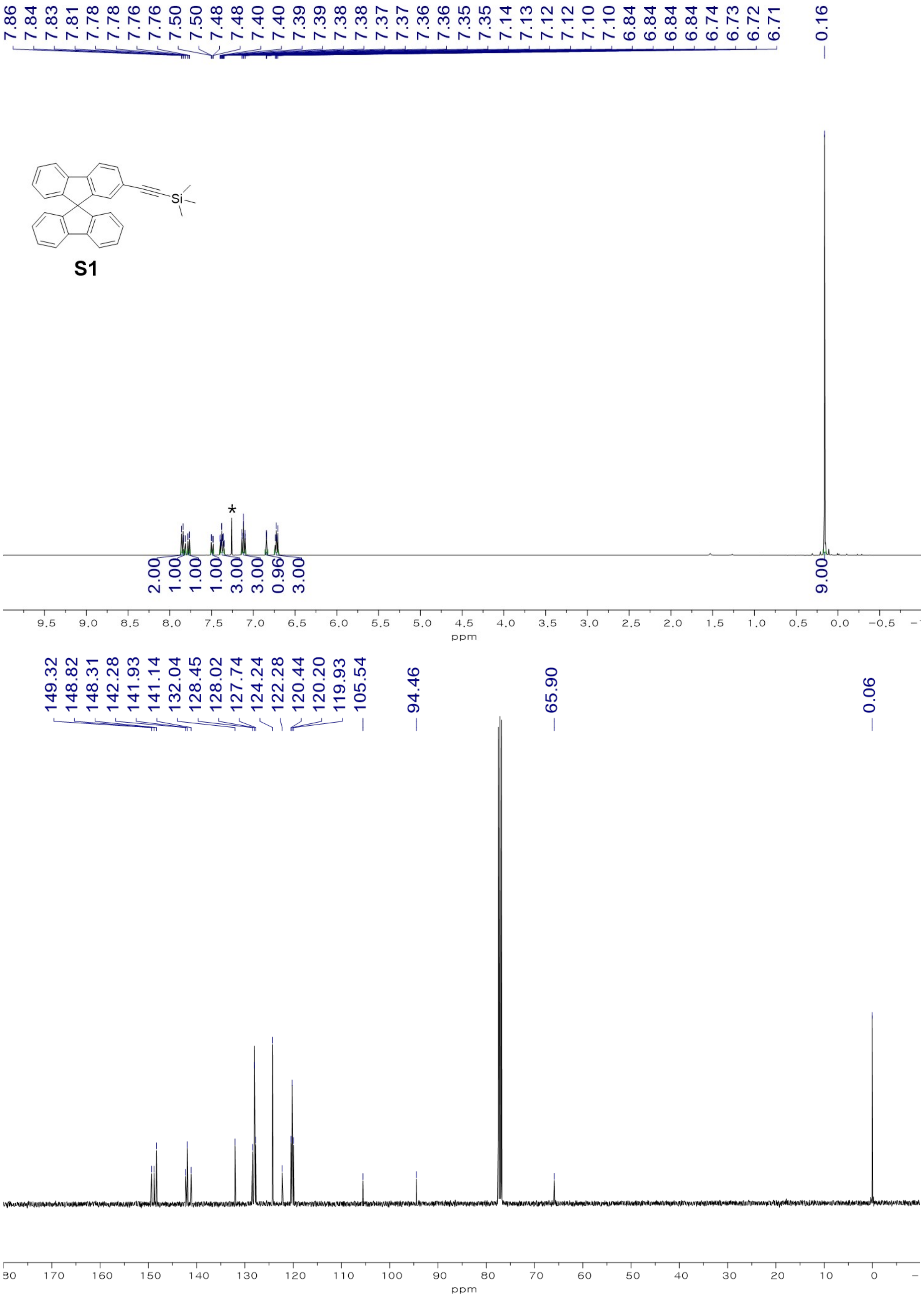

Figure S1. ^{1}H (top) and ^{13}C (bottom) NMR spectra of **S1** (∗ from residual $CHCl_3$ in $CDCl_3$)

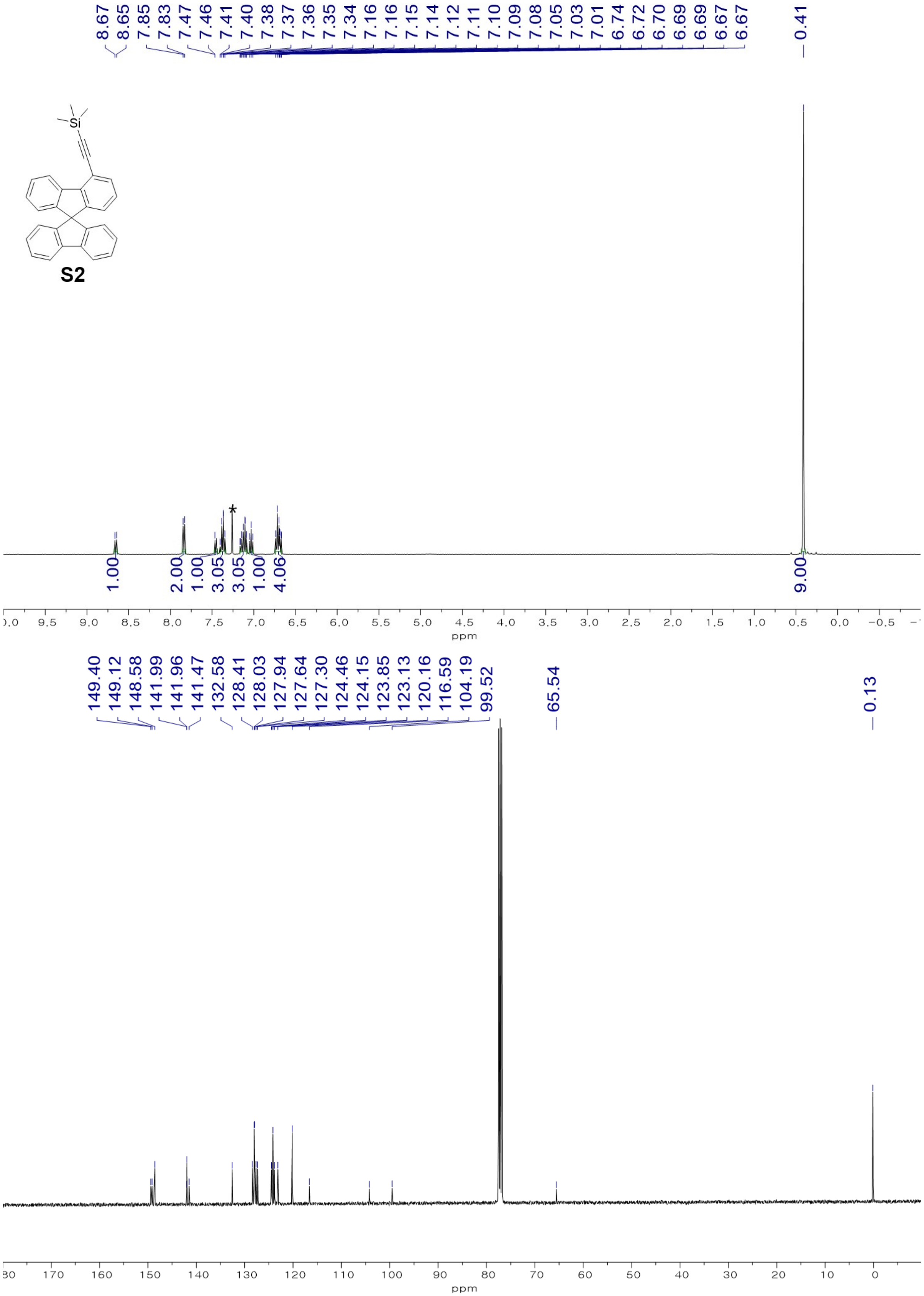

Figure S2. ^{1}H (top) and ^{13}C (bottom) NMR spectra of **S2** (∗ from residual $CHCl_3$ in $CDCl_3$)

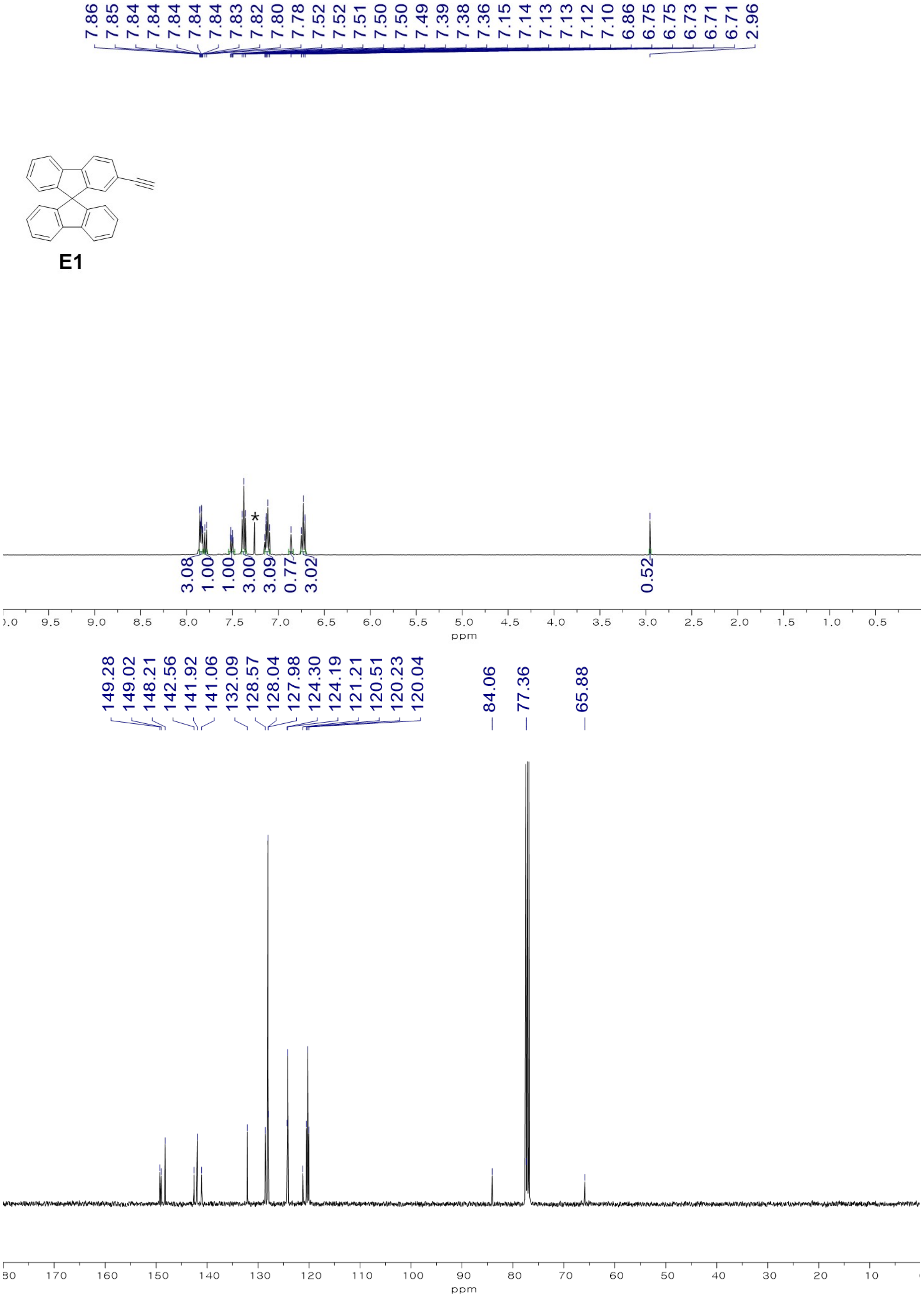

Figure S3. ^{1}H (top) and ^{13}C (bottom) NMR spectra of **E1** (∗ from residual $CHCl_3$ in $CDCl_3$)

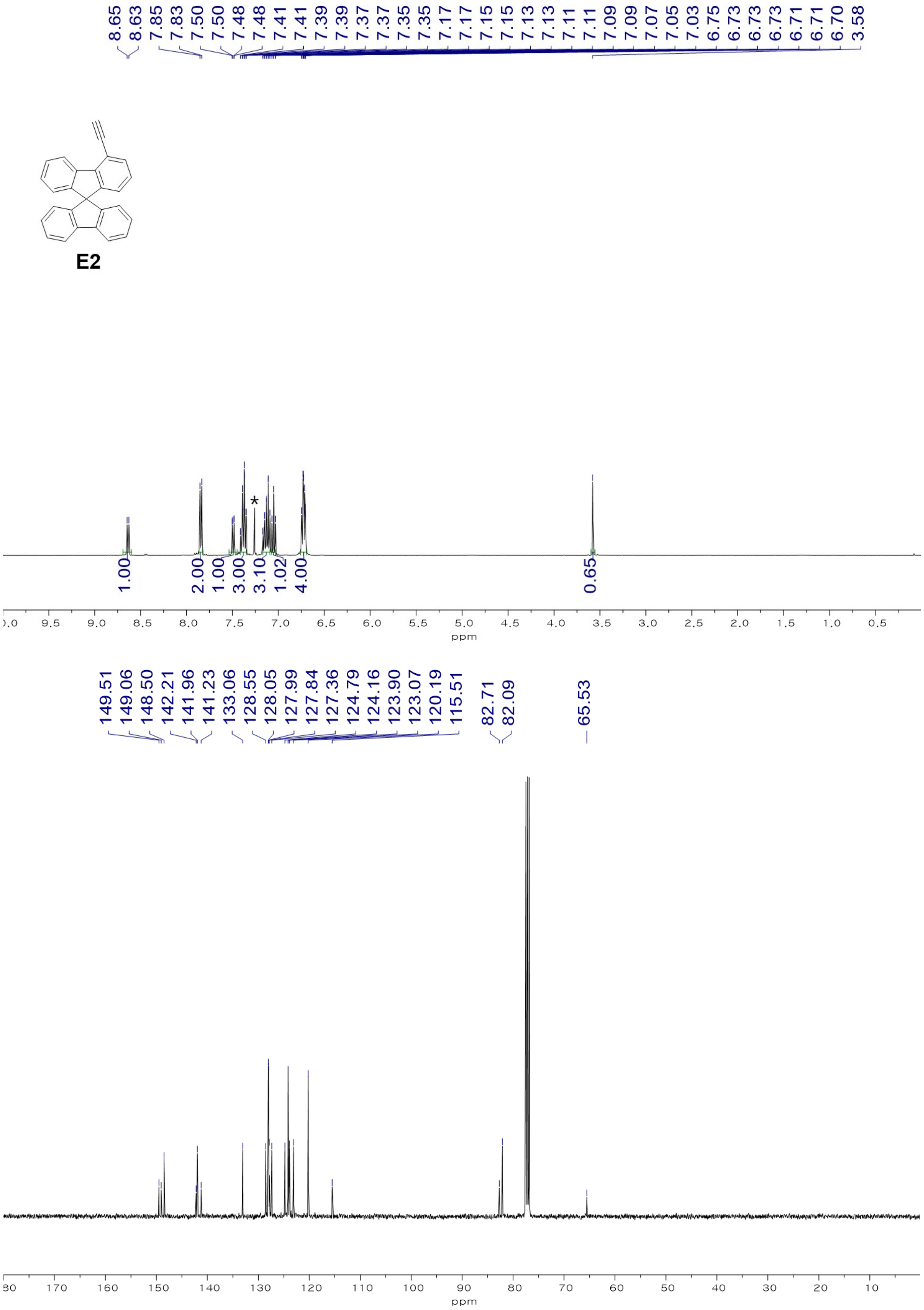

Figure S4. ^{1}H (top) and ^{13}C (bottom) NMR spectra of **E2** (∗ from residual $CHCl_3$ in $CDCl_3$)

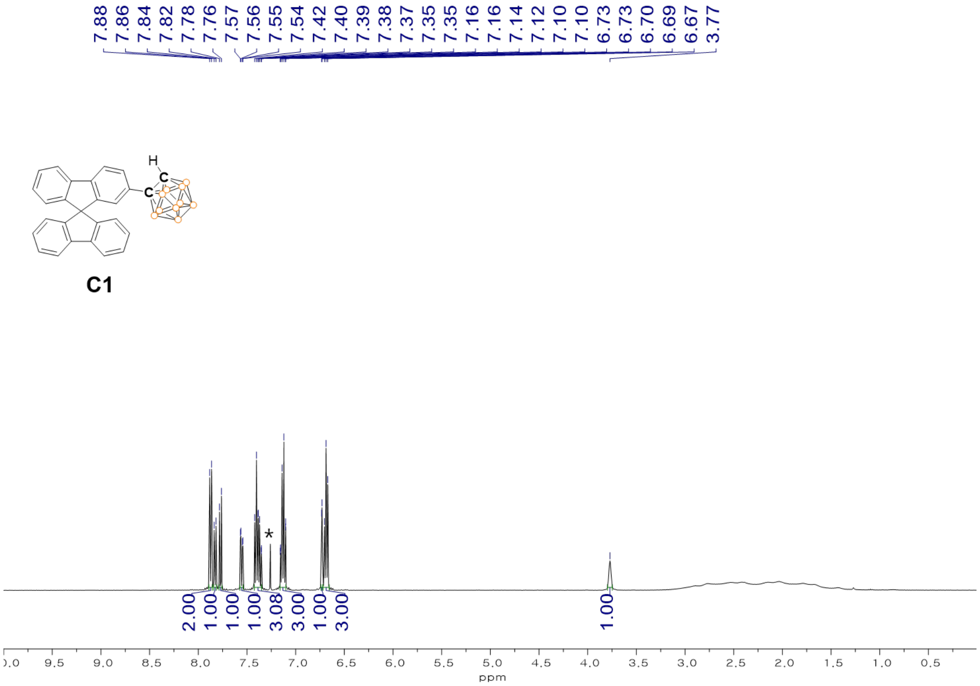

C1

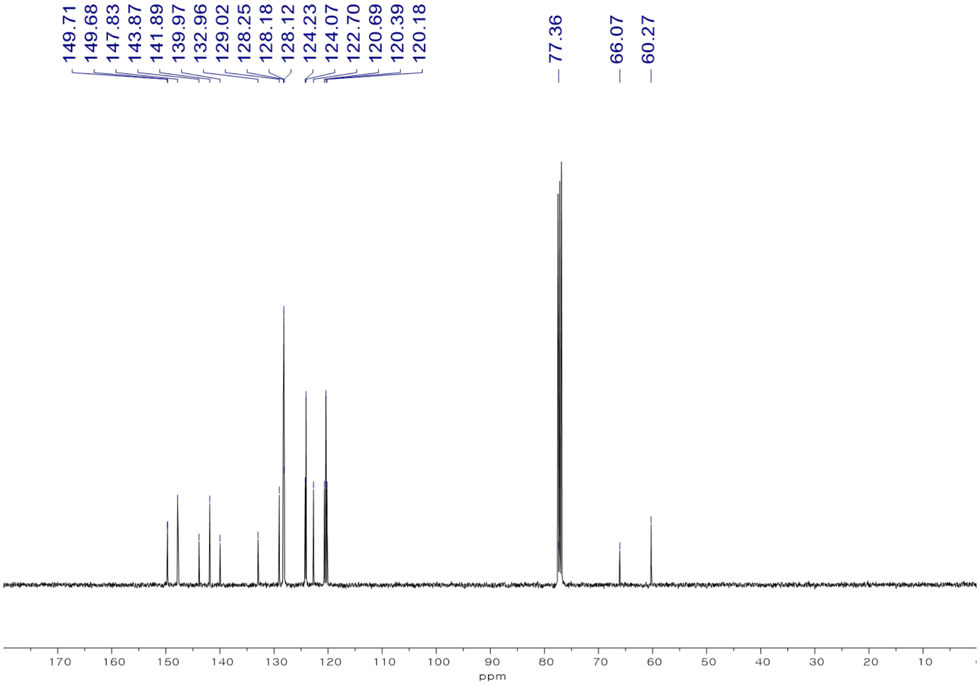

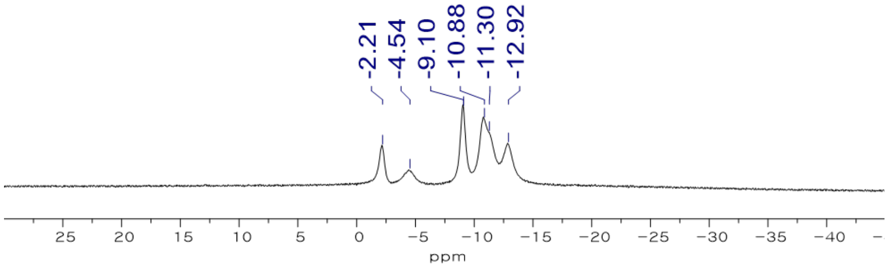

Figure S5. ^{1}H (top), ^{13}C (middle) and ^{1}H decoupled ^{11}B NMR spectra of **C1** (∗ from residual $CHCl_3$ in $CDCl_3$)

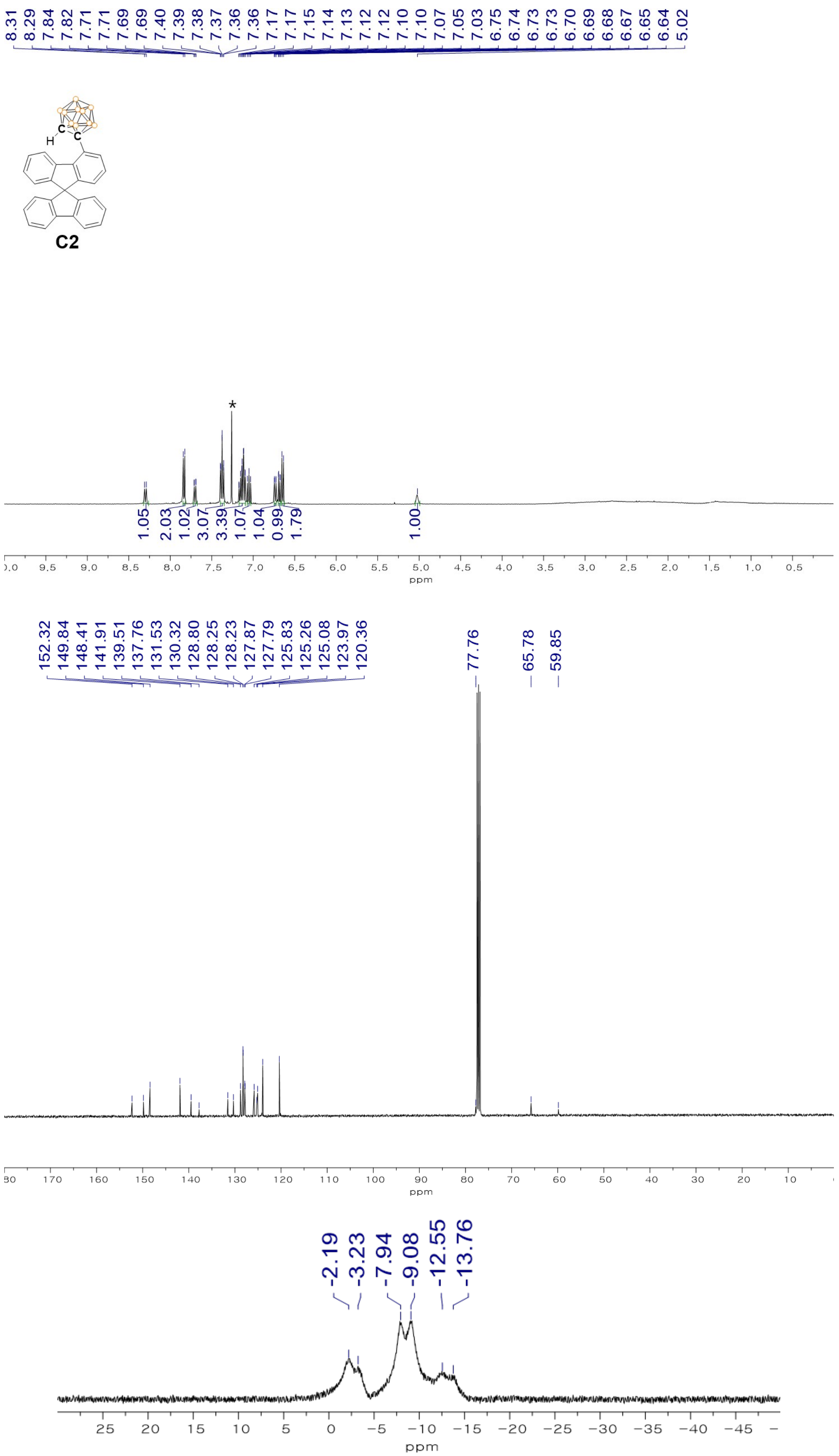

Figure S6. ^{1}H (top), ^{13}C (middle) and ^{1}H decoupled ^{11}B NMR spectra of **C2** ($*$ from residual $CHCl_3$ in $CDCl_3$)

Table S1. Crystallographic data and parameters for **C1**.

Compound	**C1**
Formula	$C_{27}H_{26}B_{10}$
Formula weight	458.58
Crystal system	Orthorhombic
Space group	Pbca
a (Å)	13.6476(12)
b (Å)	18.5275(16)
c (Å)	20.483(2)
α (°)	90
β (°)	90
γ (°)	90
V (Å^3)	5179.3(8)
Z	8
ρ_{calc}(g cm^{-3})	1.176
μ (mm^{-1})	0.061
F(000)	1904
T (K)	153(2)
Scan mode	*multi-scan*
hkl range	$-18 < h < 17$, $-24 < k < 23$, $-27 < l < 27$
Measd reflns	62733
Unique reflns [R_{int}]	6401 [0.1352]
Reflns used for refinement	6401
Refined parameters	367
$R_1{}^a$ (I > 2σ(I))	0.0694
$wR_2{}^b$ all data	0.1730
GOF on F^2	1.081
ρ_{fin} (max/min) (e Å^{-3})	0.277, −0.299

$^a R_1 = \sum||Fo| - |Fc||/\sum|Fo|$. $^b wR_2 = \{[\sum w(Fo^2 - Fc^2)^2]/[\sum w(Fo^2)^2]\}^{1/2}$.

Table S2. Selected bond lengths (Å) and angles (°) for **C1**

bond lengths (Å)			
C1–C2	1.662(3)	C14–C15	1.532(3)
C2–C3	1.507(3)	C15–C16	1.522(3)
C7–C15	1.522(3)	C15–C23	1.525(3)
angles (°)			
C7–C15–C16	114.56(16)	C14–C15–C16	114.18(16)
C7–C15–C14	101.14(16)	C14–C15–C23	113.18(16)
C7–C15–C23	113.09(16)	C16–C15–C23	101.26(16)

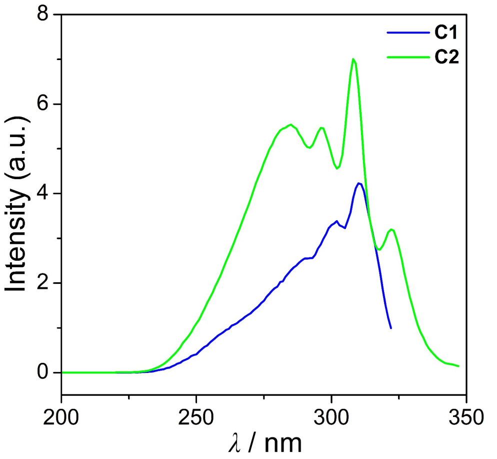

Figure S7. Excitation graphs in THF (30 μM) of **C1** and **C2**.

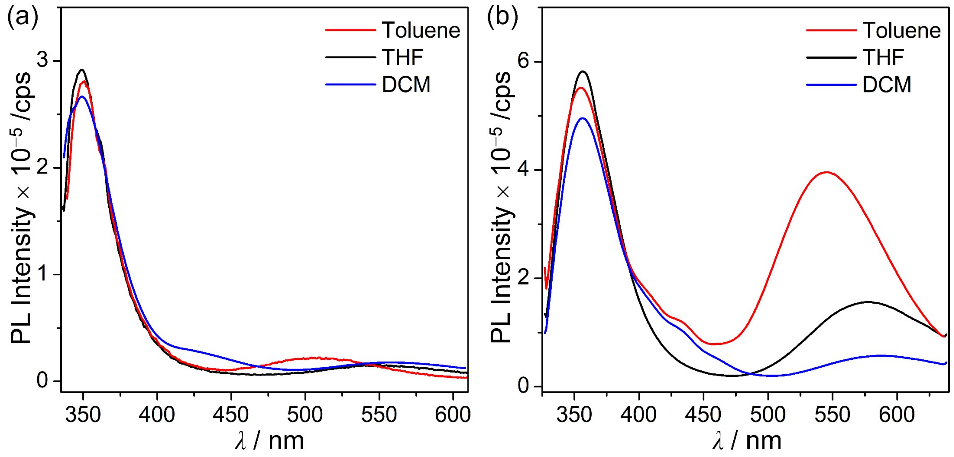

Figure S8. PL spectra of (a) **C1** and (b) **C2** in various organic solvents (30 μM, λ_{ex} = 310 nm for **C1** and 322 nm for **C2**).

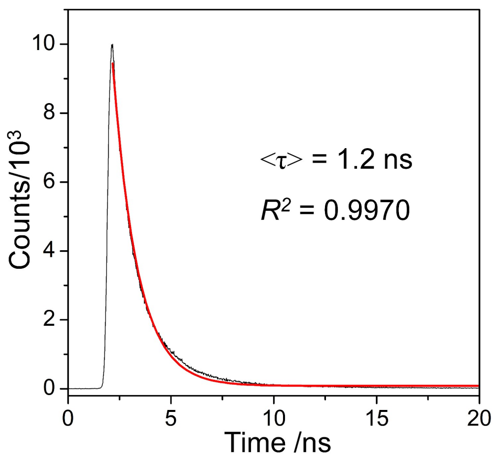

Figure S9. Emission decay curve for **C1** in film state (5 wt% doped in PMMA) detected at 490 nm at 298 K. The red-line is its single exponential fitting curve.

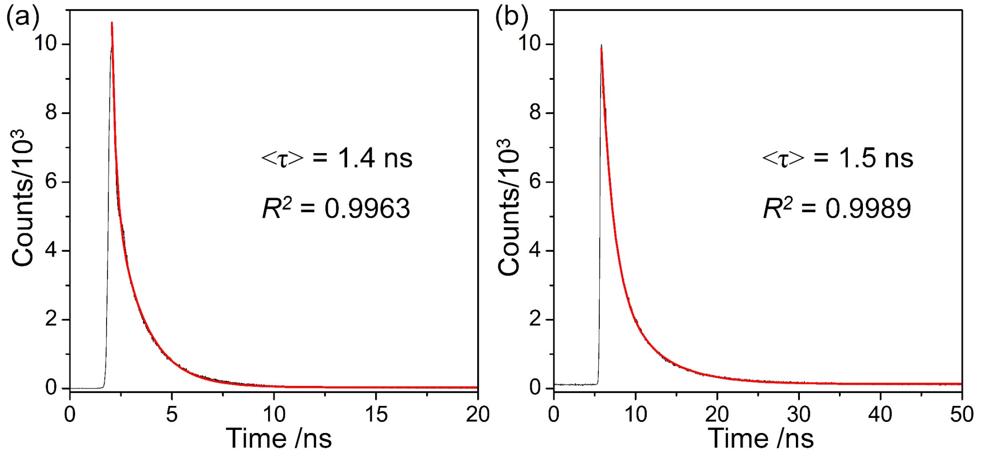

Figure S10. Emission decay curves for **C2** in (a) THF (3.0×10^{-5} M) detected at 577 nm and (b) film state (5 wt% doped in PMMA) detected at 539 nm at 298 K. Each red-line is its single exponential fitting curve.

Computational details

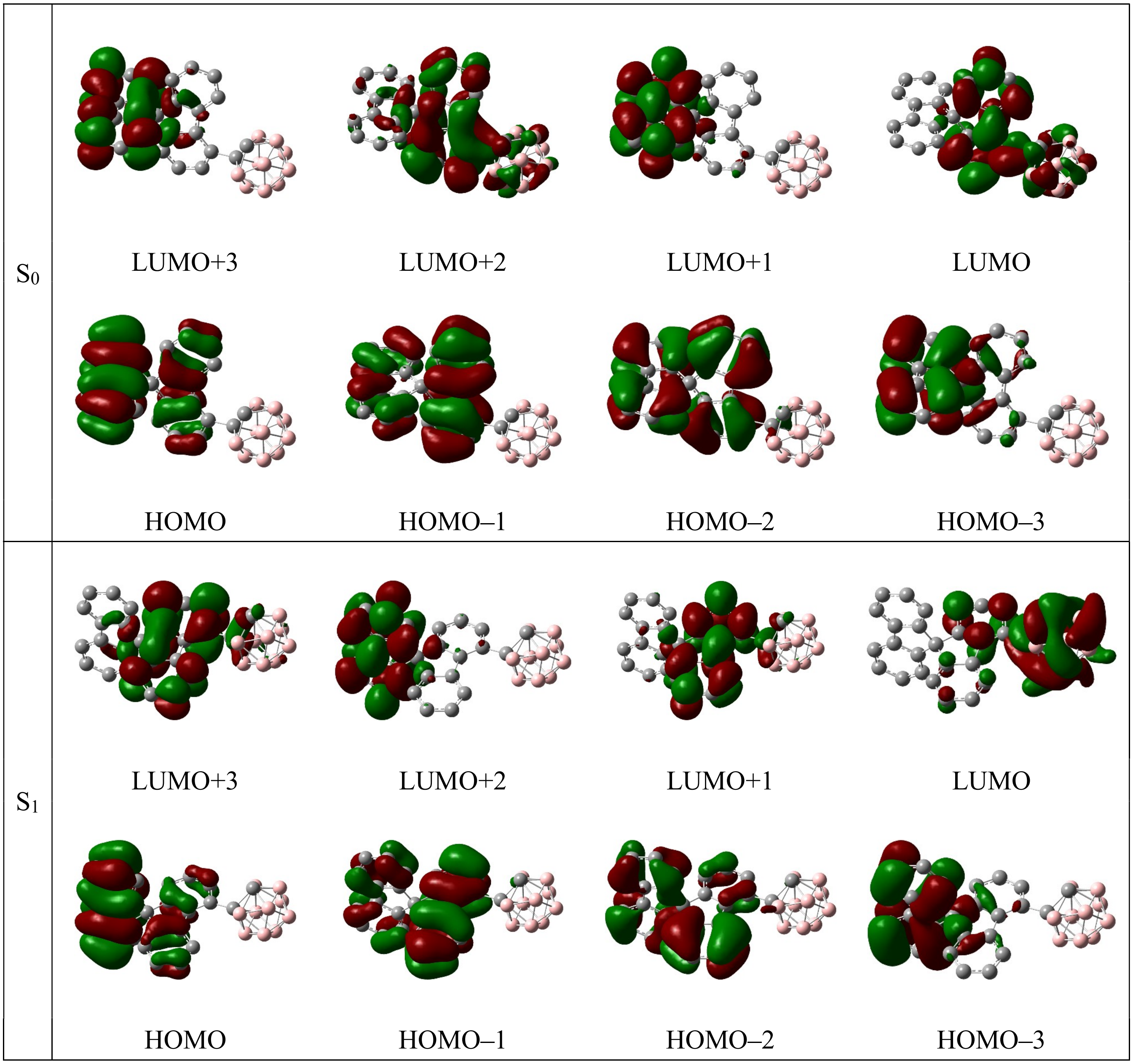

Figure S11. The selected frontier orbitals of **C1** from B3LYP calculations (Isovalue = 0.04 a.u.) at the ground state (S_0) and first singlet excited state (S_1) optimized geometries in THF.

Table S3. Computed absorption wavelengths (λ_{calc} in nm) and oscillator strengths ($f_{calc.}$) for **C1** from TD-B3LYP calculations using the B3LYP geometries at the ground state (S_0) and first singlet excited state (S_1) optimized geometries in THF

state	λ_{calc} (/nm)	f_{calc}	Major contribution
			S_0
1	316.26	0.1079	HOMO → LUMO (97.7%)
2	287.61	0.0277	HOMO−1 → LUMO (95.7%)
3	284.99	0.0491	HOMO → LUMO+1 (85.4%)
4	279.93	0.0084	HOMO−2 → LUMO (11.2%)
			HOMO → LUMO+2 (74.2%)
5	271.19	0.0233	HOMO−2 → LUMO+1 (7.8%)
			HOMO → LUMO+3 (73.4%)
			S_1
1	576.80	0.2194	HOMO → LUMO (99.8%)
2	427.90	0.0576	HOMO−1 → LUMO (99.2%)
3	415.74	0.1309	HOMO−1 → LUMO+1 (91.2%)
4	404.18	0.0023	HOMO−2 → LUMO (98.8%)
5	371.67	0.0098	HOMO−3 → LUMO (87.7%)

Table S4. Molecular orbital energies (in eV) and molecular orbital distributions (in %) of **C1** at the ground state (S_0) and first singlet excited state (S_1) optimized geometries in THF

	E (eV)	carborane	car-fluorene	fluorene
		S_0		
LUMO+2	−0.82	7.0	84.8	8.2
LUMO+1	−1.03	0.0	1.5	98.5
LUMO	−1.46	15.9	83.4	0.7
HOMO	−5.94	0.8	27.5	71.7
HOMO−1	−6.24	2.7	65.4	31.9
HOMO−2	−6.75	0.5	48.6	50.9
		S_1		
LUMO+2	−1.19	2.2	21.7	76.1
LUMO+1	−1.19	7.6	70.2	22.2
LUMO	−3.50	82.9	17.0	0.1
HOMO	−5.76	0.5	8.6	90.9
HOMO−1	−6.34	7.8	76.7	15.5
HOMO−2	−6.84	1.3	49.6	49.0

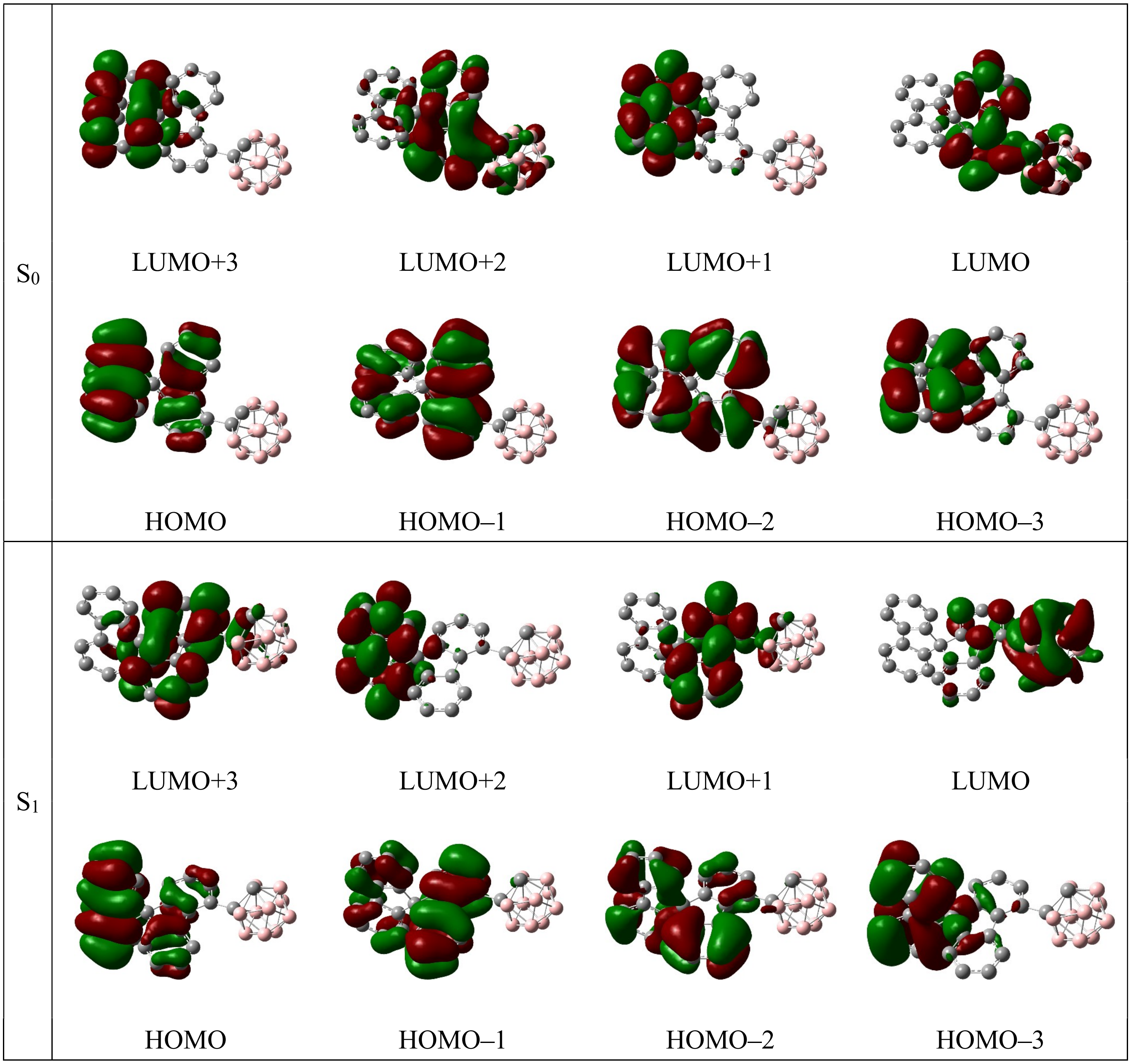

Figure S12. The selected frontier orbitals of **C2** from B3LYP calculations (Isovalue = 0.04 a.u.) at the ground state (S_0) and first singlet excited state (S_1) optimized geometries in THF.

Table S5. Computed absorption wavelengths (λ_{calc} in nm) and oscillator strengths ($f_{calc.}$) for **C2** from TD-B3LYP calculations using the B3LYP geometries at the ground state (S_0) and first singlet excited state (S_1) optimized geometries in THF

state	λ_{calc} (/nm)	f_{calc}	Major contribution
			S_0
1	324.89	0.1440	HOMO → LUMO (97.4%)
2	293.09	0.0999	HOMO−1 → LUMO (91.7%)
3	284.25	0.1715	HOMO−1 → LUMO+1 (6.8%)
			HOMO → LUMO+1 (81.6%)
4	281.65	0.0190	HOMO → LUMO+2 (84.9%)
5	270.52	0.0321	HOMO → LUMO+1 (7.9%)
			HOMO → LUMO+3 (71.3%)
			S_1
1	571.55	0.1964	HOMO → LUMO (98.6%)
2	467.69	0.0034	HOMO−1 → LUMO (97.8%)
3	432.64	0.0280	HOMO−1 → LUMO (93.9%)
4	407.40	0.2902	HOMO−1 → LUMO+1 (93.1%)
5	391.12	0.0019	HOMO−2 → LUMO (92.6%)

Table S6. Molecular orbital energies (in eV) and molecular orbital distributions (in %) of **C2** at the ground state (S_0) and first singlet excited state (S_1) optimized geometries in THF

	E (eV)	carborane	car-fluorene	fluorene
		S_0		
LUMO+2	−0.91	12.1	77.8	10.1
LUMO+1	−1.04	0.6	3.2	96.2
LUMO	−1.57	15.3	83.4	1.3
HOMO	−5.96	0.1	20.9	79.0
HOMO−1	−6.31	1.0	71.3	27.7
HOMO−2	−6.76	2.7	46.8	50.5
		S_1		
LUMO+2	−1.17	0.2	4.0	95.9
LUMO+1	−1.28	5.8	90.6	3.7
LUMO	−3.65	82.1	17.7	0.1
HOMO	−5.78	0.0	13.6	86.4
HOMO−1	−6.31	2.0	77.5	20.5
HOMO−2	−6.79	1.6	60.2	38.2

Table S7. Cartesian coordinates of the ground state (S_0) fully optimized geometry in THF of **C1** from B3LYP calculations (in Å)

Atom	x	y	z
C	3.410212	-0.884687	-0.660174
C	3.140435	0.574536	0.055778
B	4.579645	-1.811619	0.147613
B	4.938350	-0.997814	-1.394601
B	5.133242	-0.764413	1.474765
B	6.079304	-0.876093	-0.039229
B	5.830002	0.706495	0.740978
B	5.706612	0.561498	-1.037643
B	4.185249	0.743032	1.411199
B	4.539156	1.551773	-0.134700
B	3.995857	0.508240	-1.463082
B	3.422926	-0.818162	1.055324
H	4.438085	-2.985471	0.177304
H	5.030738	-1.632729	-2.388220
H	7.135583	-1.415380	-0.081596
H	5.499559	-1.216004	2.508634
H	2.501013	-1.257849	1.642869
H	3.417335	0.886610	-2.418274
H	6.483368	1.059914	-1.782739
H	6.705961	1.316981	1.259592
H	3.782033	1.368191	2.330470
H	4.414086	2.720635	-0.244370
C	1.734083	1.128317	0.035577
C	0.620772	0.269956	0.030176
C	1.530617	2.518045	0.035949
C	-0.656546	0.809432	0.011893
H	0.743384	-0.807453	0.052447
C	0.245410	3.059302	0.024974
H	2.381849	3.186721	0.046480
C	-1.997426	0.068743	-0.001124
C	-0.855336	2.202020	0.008823
H	0.116788	4.137145	0.027288
C	-2.983481	1.241426	-0.014741
C	-2.156656	-0.890153	-1.186764
C	-2.183492	-0.884515	1.185200
C	-2.298352	2.471902	-0.008325
C	-4.373189	1.205444	-0.031130
C	-2.396356	-2.202370	-0.735291
C	-2.091243	-0.606716	-2.546099
C	-2.413417	-2.198796	0.734705
C	-2.149157	-0.594495	2.544289
C	-3.003641	3.677866	-0.018212
C	-5.078772	2.413892	-0.041182
H	-4.905149	0.258563	-0.036045
C	-2.571556	-3.242564	-1.651214
C	-2.267252	-1.650317	-3.462247
H	-1.907659	0.405315	-2.895496
C	-2.610961	-3.234360	1.651322
C	-2.347037	-1.633530	3.461096
H	-1.972698	0.419023	2.892979
C	-4.399509	3.638402	-0.034772
H	-2.480707	4.629698	-0.013313
H	-6.164431	2.401465	-0.054055
C	-2.505059	-2.955942	-3.017077
H	-2.756603	-4.257754	-1.312329
H	-2.219239	-1.444493	-4.527296
C	-2.575861	-2.941093	3.016910
H	-2.789376	-4.250988	1.313208
H	-2.323098	-1.422622	4.525949
H	-4.963737	4.566031	-0.042750
H	-2.639526	-3.754307	-3.740793
H	-2.727748	-3.735770	3.741225
H	2.545088	-1.348604	-1.114319

Table S8. Cartesian coordinates of the first excited state (S_1) fully optimized geometry in THF of **C1** from B3LYP calculations (in Å)

Atom	x	y	z
C	4.384283	0.008916	-1.642376
C	3.026388	0.574577	0.234862
B	5.211327	-1.293073	-1.129294
B	5.888502	0.359265	-1.136395
B	4.723549	-1.669404	0.529079
B	6.233355	-0.741394	0.215526
B	5.240364	-0.328539	1.607774
B	5.811154	0.981140	0.518241
B	3.517751	-0.599787	1.245036
B	4.203630	1.072922	1.237701
B	4.379307	1.328245	-0.592545
B	3.458507	-0.917276	-0.583304
H	5.465163	-2.151216	-1.913322
H	6.669243	0.786238	-1.925967
H	7.327448	-1.189248	0.371966
H	4.739212	-2.806042	0.888636
H	2.572277	-1.576256	-1.025320
H	4.208665	2.416079	-1.041744
H	6.621936	1.781062	0.870051
H	5.647240	-0.490702	2.717334
H	2.708691	-1.019589	2.010242
H	3.925044	1.945429	1.997223
C	1.667247	1.127584	0.165200
C	0.545084	0.273022	0.123771
C	1.442510	2.524624	0.135965
C	-0.727591	0.818035	0.053742
H	0.691003	-0.801408	0.147106
C	0.162024	3.067015	0.066927
H	2.297933	3.190180	0.170892
C	-2.067631	0.075555	-0.005484
C	-0.941190	2.209697	0.022722
H	0.031129	4.144986	0.049246
C	-3.067387	1.234166	-0.071392
C	-2.159105	-0.891009	-1.177291
C	-2.283842	-0.864974	1.170723
C	-2.381490	2.468588	-0.054003
C	-4.454535	1.179874	-0.140650
C	-2.371374	-2.231331	-0.711530
C	-2.054595	-0.632477	-2.532837
C	-2.449220	-2.215246	0.714633
C	-2.323922	-0.576583	2.523825
C	-3.101500	3.664170	-0.107280
C	-5.170098	2.381571	-0.193744
H	-4.978738	0.228628	-0.154224
C	-2.470301	-3.309927	-1.620602
C	-2.154553	-1.707538	-3.421556
H	-1.894454	0.373980	-2.902068
C	-2.652115	-3.272710	1.631166
C	-2.523981	-1.631212	3.419950
H	-2.200283	0.437535	2.886033
C	-4.496391	3.608916	-0.176899
H	-2.590189	4.621872	-0.095070
H	-6.253875	2.361066	-0.248506
C	-2.360196	-3.035921	-2.968177
H	-2.627396	-4.321984	-1.265870
H	-2.071423	-1.525265	-4.487463
C	-2.686942	-2.968742	2.976376
H	-2.776779	-4.291816	1.283863
H	-2.554868	-1.425385	4.484374
H	-5.066340	4.532240	-0.218754
H	-2.429722	-3.836729	-3.695325
H	-2.839360	-3.752702	3.709208
H	4.063750	0.136723	-2.667890

Table S9. Cartesian coordinates of the ground state (S_0) fully optimized geometry in THF of **C2** from B3LYP calculations (in Å)

Atom	x	y	z
C	-3.440676	-0.346703	0.877136
C	-3.064311	0.353808	-0.549608
B	-4.887155	-1.239540	0.821739
B	-4.788595	0.331511	1.655003
B	-5.513942	-0.960428	-0.819551
B	-6.142000	-0.010525	0.558499
B	-5.790863	0.793228	-0.994381
B	-5.342744	1.590212	0.539586
B	-4.329286	0.052918	-1.683710
B	-4.231297	1.616769	-0.841364
B	-3.612057	1.346871	0.792425
B	-3.790349	-1.213646	-0.574704
H	-4.950412	-2.238523	1.452050
H	-4.781078	0.366105	2.837206
H	-7.242919	-0.159550	0.975509
H	-6.149168	-1.784848	-1.388453
H	-3.106380	-2.116527	-0.895590
H	-2.798817	2.016523	1.321993
H	-5.854980	2.588238	0.925032
H	-6.634627	1.229724	-1.706014
H	-4.024770	-0.035659	-2.822729
H	-3.897091	2.596186	-1.406980
C	0.951340	1.468099	-1.778739
C	-1.438889	1.476978	-2.015368
C	0.808327	0.497093	-0.800241
H	1.935624	1.845502	-2.037401
C	-1.614406	0.533881	-0.984900
H	-2.311983	1.891452	-2.498843
C	1.958878	-0.093628	0.004001
C	-0.444128	-0.057406	-0.425852
C	1.243874	-1.175890	0.790895
C	3.121467	-0.616462	-0.846248
C	2.669911	0.949384	0.878671
C	-0.140509	-1.189964	0.507913
C	1.835511	-2.103962	1.639064
C	4.321533	0.034913	-0.502228
C	3.100010	-1.588847	-1.839541
C	4.042027	1.003525	0.567687
C	2.130752	1.767682	1.864236
C	-0.896625	-2.253356	1.028355
C	1.051544	-3.112023	2.202436
H	2.901416	-2.054829	1.840903
C	5.512724	-0.288066	-1.156712
C	4.294378	-1.910894	-2.494470
H	2.174964	-2.092164	-2.105840
C	4.885200	1.884529	1.249016
C	2.977308	2.650008	2.545652
H	1.072097	1.727068	2.104316
C	-0.302333	-3.193600	1.873642
H	-1.928328	-2.410159	0.755955
H	1.499028	-3.845861	2.865363
C	5.489121	-1.265316	-2.154873
H	6.442760	0.209547	-0.897687
H	4.293483	-2.668183	-3.272600
C	4.342002	2.706641	2.239625
H	5.944999	1.932555	1.016178
H	2.571034	3.295880	3.318150
H	-0.908025	-4.004840	2.265405
H	6.407139	-1.526888	-2.672610
H	4.985146	3.396621	2.777663
C	-0.189538	1.926000	-2.429062
H	-0.119707	2.654786	-3.229719
H	-2.604922	-0.687552	1.468120

Table S10. Cartesian coordinates of the first excited state (S_1) fully optimized geometry in THF of **C2** from B3LYP calculations (in Å)

Atom	x	y	z
C	-4.342854	1.366195	-1.083853
C	-2.980671	0.235254	0.569284
B	-5.820260	1.444369	-0.410233
B	-5.339037	0.188237	-1.585868
B	-5.735796	0.723543	1.200626
B	-6.357413	-0.234933	-0.190397
B	-5.397285	-1.030030	1.045750
B	-4.991179	-1.295340	-0.686181
B	-4.160358	0.088057	1.687494
B	-3.709444	-1.211413	0.525659
B	-3.579869	-0.133903	-1.018798
B	-4.226478	1.554127	0.569252
H	-6.502201	2.391752	-0.638851
H	-5.648590	0.152651	-2.733834
H	-7.504425	-0.526500	-0.335554
H	-6.443520	1.144637	2.062267
H	-3.890548	2.612256	0.991006
H	-2.790223	-0.376745	-1.868694
H	-5.171582	-2.343997	-1.223983
H	-5.877548	-1.862921	1.751592
H	-3.823915	0.144463	2.826763
H	-3.052616	-2.167063	0.781173
C	1.005541	1.637939	1.621081
C	-1.394581	1.607449	1.852182
C	0.867167	0.597575	0.710673
H	1.981724	2.043016	1.867019
C	-1.577895	0.578161	0.901533
H	-2.276216	2.027833	2.318025
C	2.023408	-0.073963	-0.015468
C	-0.382576	0.020757	0.349816
C	1.314245	-1.167457	-0.790160
C	2.811878	0.881014	-0.898880
C	3.110881	-0.599227	0.907932
C	-0.085276	-1.116136	-0.558550
C	1.906441	-2.129033	-1.602604
C	4.193528	0.889391	-0.520476
C	2.364987	1.674295	-1.945773
C	4.375282	-0.012495	0.580108
C	3.008709	-1.513444	1.946400
C	-0.876834	-2.110723	-1.149977
C	1.095880	-3.093561	-2.198505
H	2.980722	-2.133734	-1.761036
C	5.123227	1.701997	-1.201622
C	3.294352	2.474937	-2.610779
H	1.321715	1.676974	-2.240901
C	5.537455	-0.351118	1.303747
C	4.165534	-1.839508	2.655796
H	2.056816	-1.963887	2.204505
C	-0.284127	-3.081082	-1.958980
H	-1.939865	-2.149621	-0.985510
H	1.532453	-3.856087	-2.835390
C	4.663130	2.487592	-2.241142
H	6.169154	1.708717	-0.916498
H	2.966248	3.104800	-3.430602
C	5.420025	-1.262769	2.335680
H	6.495335	0.092098	1.056013
H	4.107572	-2.550550	3.472903
H	-0.912417	-3.843079	-2.409986
H	5.350608	3.124709	-2.786216
H	6.293213	-1.545397	2.913010
C	-0.153025	2.127609	2.214495
H	-0.101619	2.923855	2.950423
H	-3.957651	2.130383	-1.745906
